# Examining the Effect of Adverbs and Onomatopoeia on Physical Movement

**DOI:** 10.3389/fpsyg.2021.723602

**Published:** 2021-09-22

**Authors:** Keisuke Irie, Shuo Zhao, Kazuhiro Okamoto, Nan Liang

**Affiliations:** ^1^Cognitive Motor Neuroscience, Department of Human Health Sciences, Graduate School of Medicine, Kyoto University, Kyoto, Japan; ^2^School of Psychology, Shenzhen University, Shenzhen, China; ^3^Department of Rehabilitation, Faculty of Health Science, Fukui Health Science University, Fukui, Japan

**Keywords:** ACE, action verbs, onomatopoeia, grounded cognition, adverbs (intensifying)

## Abstract

**Introduction:** The effect of promoting a physical reaction by the described action is called the action-sentence compatibility effect (ACE). It has been verified that physical motion changes depending on the time phase and grammatical expression. However, it is unclear how adverbs and onomatopoeia change motion simulations and subsequent movements.

**Methods:** The subjects were 35 healthy adults (11 females; mean age 21.3). We prepared 20 sentences each, expressing actions related to hands and feet. These were converted into 80 sentences (stimulus set A), with the words “Slow” or “Quick” added to the words related to the speed of movement, and 80 sentences (stimulus set B) with the words “Fast” and onomatopoeia “Satto” added. Additionally, 20 unnatural sentences were prepared for each stimulus set as pseudo sentences. Choice reaction time was adopted; subjects pressed the button with their right hand only when the presented text was correctly understood (Go no-go task). The reaction time (RTs) and the number of errors (NoE) were recorded and compared.

**Results:** As a result of a two-way repeated ANOVA, an interaction effect (body parts × words) was observed in RTs and NoE in set A. “Hand and Fast” had significantly faster RTs than “Hand and Slow” and “Foot and Fast.” Furthermore, “Hand and Fast” had a significantly higher NoE than others. In set B, the main effects were observed in both RTs and NoE. “Hand” and “Satto” had significantly faster RTs than “Foot” and “Quick,” respectively. Additionally, an interaction effect was observed in NoE, wherein “Foot and Satto” was significantly higher than “Hand and Satto” and “Foot and Quick.”

**Conclusion:** In this study, the word “Fast” promoted hand response, reaffirming ACE. The onomatopoeia “Satto” was a word that conveys the speed of movement, but it was suggested that the degree of understanding may be influenced by the body part and the attributes of the subject.

## Introduction

In sports and rehabilitation, motor learning is promoted by explaining how the movement needs to be performed to the individual and providing knowledge of results (KR). Although KR is useful for motor correction, studies have shown that excessive dependence on KR negatively impacts subsequent performance (Badets and Blandin, 2010; Chiviacowsky et al., 2010) by making it less likely for internal feedback to develop. Therefore, it is necessary to promote internal feedback by gradually reducing the frequency of KR. Recent suggestions state that a combination of coaching and exercise effectively improves both athletic and psychosocial performance (Kivelä et al., 2014; Ovans et al., 2018). Thus, language can be used to affect the movements of individuals in many situations.

“Onomatopoeia” is a word that imitates the sounds made by people, animals, or nature. According to the Oxford English Dictionary, “onomatopoeia” is defined as “the formation of words from sounds associated with the thing named.” These are well-established words with a long history, dating back at least four centuries (Assaneo et al., 2011). The term onomatopoeia is also used in motion instruction to describe the motion’s force, speed, and timing. In Japanese, examples include “Gutto” (Putting in a moment of effort), “Satto” (“In a flash”), and “Sotto (Doing something quietly).” It allows for the concise expression of meanings and images that are otherwise difficult to express; consequently, it has been documented as an important language learning and communication tool for young children (Laing, 2017). Onomatopoeia falls under the expressive aspect of communication, as it often conveys extremely vague impressions that are difficult to express purely as propositions (Blakemore, 2008, 2011, 2014). Sasamoto and Jackson (2016) have shown that communicators can use any available tools and that onomatopoeia results from attempts to replicate sensory experiences, especially through the use of similarity. However, few papers have examined whether onomatopoeia accurately conveys the intended information using experimental methods.

In the last two decades, many studies have investigated the association between sentence comprehension and sensorimotor representation. Grounded cognition proposes that modal simulations, body states, and situated actions underlie cognition, and the representation of concepts activates the same sensory-motor modalities that recognize and act on those concepts (Barsalou, 1999, 2008, 2010; Glenberg and Kaschak, 2002; Gallese and Lakoff, 2005). This simulation reflects the meaning of the words and the meaning and grammar of the whole sentence. For example, negative sentences undergo a two-step process in which the affirmative sentence is first simulated and then negated (Kaup et al., 2006, 2007). Furthermore, some studies have shown that the progressive form facilitates the response, but the past tense of the verb does not (Madden and Therriault, 2009; Bergen and Wheeler, 2010). Behavioral experiments have repeatedly reported that when an active sentence is judged to be meaningful, the physical movement or action associated with that sentence is promoted (Dudschig et al., 2012; Diefenbach et al., 2013; Ibáñez et al., 2013). Specifically, subjects pressed one of two buttons placed near or far from a given body after determining whether a sentence made sense. In some cases, the sentence included moving the hand closer to or further away from the body. For example, the sentence “he took off his glasses” represented the action of “moving his hands away from his body.” Conversely, the sentence “he put on his glasses” represented the action of “moving his hands closer to his body.” When the action described in a sentence is consistent with the action the subject must perform to respond to that sentence, the response is faster than when the actions are not consistent (Glenberg and Kaschak, 2002; Zwaan et al., 2012). This behavioral effect, known as the action-sentence compatibility effect (ACE), has been observed in multiple languages, including English (Borreggine and Kaschak, 2006), French (Boulenger et al., 2006), Italian (Glenberg et al., 2008), and Japanese (Awazu, 2011). Thus, using ACE, it is possible to investigate the influence of grammatical aspects of a sentence, in addition to the nouns and verbs used. We use the adverbs “Slow” or “Quick” when referring to the speed of movement in our daily lives. However, the effects of adverbs used in motion commands and onomatopoeia with similar meanings on physical responses have not been tested.

Therefore, the objectives of this study are as follows: first, to examine whether the words “Slow” and “Fast,” which describe the speed of the action indicated by the verb, affect the simulation of movement. In other words, we expected that words related to hand movement would facilitate both “Slow” and “Fast” hand responses, while words related to foot movement would not produce ACE; thus, it would not change the results. Second, we tested the usefulness of the onomatopoeic word “Satto” to promote speed of movement. We hypothesized that the reaction time would be shorter for “Satto” than for “Quick” in words related to hand movements. To clarify these hypotheses, we conducted a single experiment consisting of two consecutive sub-experiments (stimulus set A and stimulus set B).

## Materials and Methods

### Participants

This cross-sectional study included 35 healthy students as participants (11 females, mean age: 21.3 ± 2.4 years) from Kyoto University. All participants were informed about the procedures involved in the experiments, and those who provided their informed consent were included. The exclusion criteria were as follows: (1) those who were not native Japanese speakers and (2) those who could not perform reading comprehension or press a button, owing to cognitive or motor difficulties [IQ70 or higher was confirmed by the Japanese Adult Reading Test (Matsuoka et al., 2006)]. In addition, right-handedness was assessed using the Edinburgh Handedness Questionnaire (Oldfield, 1971). The study was approved by the Ethics Committee of Kyoto University Graduate School and the Faculty of Medicine (approval number R2188-1).

### Apparatus

The experiments were conducted using a computer (Panasonic, CF-SV) and a 17-inch display monitor (MITSUBISHI and RDT20IWM). Software (E-Prime 3.0: Psychology Software Tools, Inc.) was used to control stimulus presentation and record participants’ responses. First, participants placed their right index finger on a mark 20 cm away from the keyboard. Next, a sentence was presented at random. The participant was then instructed to press the button only if they understood the sentence.

### Stimulus

We prepared 20 types of sentences expressing actions related to each hand and foot in Japanese and converted them into two different ones: one that used the word “Fast” (e.g., “picking it up fast,” “stand up fast”) and another using the word “Slow” (e.g., “picking it up slowly,” “sit down slowly”), that is, 40 sentences using the word “Fast” and 40 using the word “Slow.” In addition to these 80 sentences, we prepared 20 pseudo-sentences with a meaningless combination of nouns and verbs (e.g., “Pour hand”). These 100 sentences were designated as stimulus set A. In stimulus set B, which was created to examine the usefulness of onomatopoeia, 20 prepared sentences were converted into “Satto” (which means “in a flash” in Japanese) and “Quick,” and 80 types of sentences were created in the same manner as above. Additionally, as in set A, we prepared 20 pseudo-sentences that used the Japanese terms for “Fast” and “Quick” (“Subayaku” and “Hayaku,” respectively). “Quick” denotes actions performed with rapid velocity within a short duration, whereas “Fast” denotes actions performed with rapid velocity within a long duration. Different combinations of words were applied to a set of sentences so that the number of letters in comparison and the control groups would be the same.

### Procedure

**FIGURE 1:**
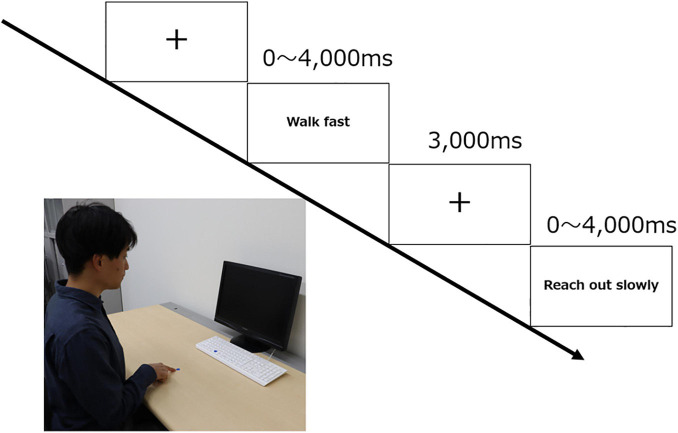
Experiment protocol followed during the study. Participants placed the index finger of their right hand on a mark 20 cm away from the keyboard. A sentence was presented at random, and the participant was instructed to press the button only if they understood the sentence.

Participants were tested after being informed about the procedures involved in the experiment and a simulated practice of the experiment had been conducted (Figure 1). The test session was composed of two 100-trial blocks, with the order of the trial blocks randomized. For each trial, a fixation cross point was first presented in the center of the screen for 3,000 ms. Subsequently, sentences related to either hand or foot movements or pseudo-sentences were presented for up to 4,000 ms. The order of the stimuli was randomized within each block. The participants took a break between blocks for approximately 3 min. In each trial, the participants were required to judge whether a stimulus sentence was meaningful or not. It was treated as a go/no-go task—they were asked to press the button only if they could understand the sentence (i.e., they were able to perform the action mentioned in the sentence). Finally, at the end of the experiment, the participants were asked which of the two terms—“Satto” or “Quick”—was easier for them to understand.

### Analysis

All statistical analyses were conducted using IBM SPSS Statistics version 26 (IBM, Armonk, NY, United States). Trials with reaction times of <300 ms and trials with reaction times greater than three standard deviations were excluded. This is because it is highly likely that the reaction time is fast considering that the sentence is not read; conversely, the reaction time could be slow for some reason other than reading. The main outcomes were reaction times (RTs) and number of errors (NoE), and a two-way repeated analysis of variance (ANOVA) was performed on words (Slow-Fast and Quick-Satto) and body parts (hand-related condition and foot-related condition). If a two-way interaction was significant, a follow-up simple main effect (i.e., assessing the effect of each independent variable at each level of the other independent variable) analysis was conducted (*p* < 0.05).

## Results

The mean RTs and NoE for stimulus sets A and B are listed in Table 1.

**TABLE 1:** Mean reaction times (RTs) and number of errors (NoE) observed in each experimental condition.

Stimulus set	Body parts (Effector)	Words (Effector)	RTs (in ms) (SE)	NoE (SE)
Set A	Hand	Slow	1710.9 (70.9)	0.5 (0.1)
		Fast	1414.5 (39.5)	1.4 (0.1)
	Foot	Slow	1749.3 (65.9)	0.7 (0.2)
		Fast	1845.7 (81.4)	0.8 (0.3)
Set B	Hand	Satto	1517.2 (43.2)	1.4 (0.2)
		Quick	1595.4 (50.6)	1.1 (0.2)
	Foot	Satto	1609.8 (54.3)	3.0 (0.5)
		Quick	1665.8 (55.1)	1.7 (0.3)

### Effect of Action-Sentence Compatibility on Reaction Times

**FIGURE 2:**
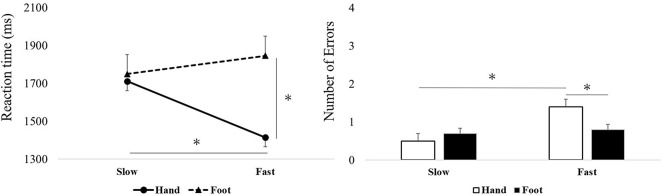
Reaction times (RTs) and number of errors (NoE) in “Slow or Fast” and “Hand or Foot” combinations. Based on the *post hoc* test, “Hand and Fast” showed significantly faster RTs, as well as a significantly greater NoE, than combinations involving “Foot” and “Slow” *p < 0.05.

The two-way repeated ANOVA conducted for the RTs for stimulus set A indicated a main effect for words, so that “Fast” produced faster RTs than “Slow” [*F*(1,34) = 79.58, *p* < 0.001 ηp^2^ = 0.70]. Furthermore, a main effect was observed for body parts, so that “Hand” produced faster RTs than “Foot” [*F*(1,34) = 12.47, *p* = 0.001, ηp^2^ = 0.27]. Moreover, the interaction effect (body parts and words) was significant [*F*(1,34) = 24.07, *p* < 0.001, ηp2 = 0.41]. In the *post hoc* test, “Hand and Fast” showed significantly faster RTs than “Hand and Slow” and “Foot and Fast” (*p* < 0.05, Figure 2).

**FIGURE 3:**
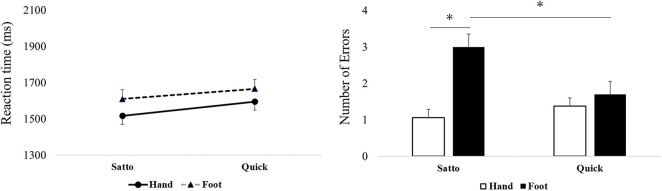
Reaction times (RTs) and number of errors (NoE) in “Satto or Quick” and “Hand or Foot” combinations. “Hand” and “Satto” led to significantly faster RTs than “Foot” and “Quick,” respectively. Additionally, “Satto” and “Foot” had more errors than “Hand,” which was higher than “Quick and Foot” *p < 0.05.

In stimulus set B, a main effect was observed for words—such that “Satto” produced faster RTs than “Quick” [*F*(1,34) = 36.34, *p* < 0.001, ηp^2^ = 0.52)—and body parts—such that “Hand” produced faster RTs than “Foot” [*F*(1,34) = 5.29, *p* = 0.028, ηp^2^ = 0.36]. However, no significant interaction effect was observed (Figure 3).

### Effect of Action-Sentence Compatibility on Number of Errors

The two-way repeated ANOVA conducted for the NoE in stimulus set A indicated a main effect for words [*F*(1,34) = 17.37, *p* < 0.001, ηp^2^ = 0.34]. Furthermore, the interaction effect was significant [*F*(1,34) = 12.11, *p* = 0.001, ηp^2^ = 0.26]. In the *post hoc* test, “Hand and Fast” was found to have a significantly higher NoE than the other conditions (*p* < 0.05, Figure 2).

In stimulus set B, no significant main effects were observed [*F*(1,34) = 0.03, *p* = 0.873]. However, the interaction effect was found to be significant [*F*(1,34) = 18.51, *p* < 0.001, ηp^2^ = 0.3]. In the *post hoc* test, NoE was significantly higher in “Foot and Satto” than “Hand and Satto” and “Foot and Quick” (*p* < 0.05, Figure 3).

### Understanding Onomatopoeia

Based on the survey conducted at the end of the experiment, it was observed that 19 participants (54.3%) felt onomatopoeia (“Satto”) was easier to understand, while 16 participants (45.7%) felt that the word “Quick” was easier to understand.

## Discussion

We hypothesized that ACE, which arises from an understanding of the words “Fast” and “Slow,” would be observed only in the case of sentences related to hand movements. Additionally, we hypothesized that the onomatopoeia “Satto” would generate a faster reaction time than “Quick” when comprehending hand-related sentences. Both hypotheses were partially confirmed; in the case of hand-related sentences, the word “Fast” led to significantly faster RTs than “Slow.” Further, although “Satto” led to significantly faster RTs than “Quick” in the case of both hand- and foot-related movements, no significant interaction effect was observed. In addition to these findings, we obtained interesting results regarding NoE; the combinations of the words “Hand and Fast” and “Foot and Satto” led to significantly higher NoE than the other combinations.

In stimulus set A, we used adverbs with opposite meanings, “Slow” and “Fast,” to test whether different adverbs showed ACE. It has been reported that familiarity with words is a factor that affects comprehension (Pulvermüller et al., 1999). According to the NTT database series, “Lexical Features of Japanese,” “Slow” has a higher degree of intimacy and mental image than “Fast” and is considered to be easier to understand. However, in this study, the reaction time of “Fast” was faster only for words related to “Hands.” This may be because the participants understood the word by simulating the action, which facilitated the same physical reaction. In other words, the ACE was confirmed for adverbs as well. Motor imagery studies have previously reported that cortical neurons are linked to task-specific activation that can inhibit or promote slow(er) motor pathways (Keller et al., 2018). It has also been suggested that both slow and fast movements involve an automated system (Cressman et al., 2010). However, in this study, the word “Fast” promoted hand response, but “Slow” did not inhibit/slow down the response. A possible explanation for this may be the influence of the experimental environment. Specifically, we can unconsciously change our movements by simply observing the surrounding objects and environment (McDannald et al., 2018). This concept is called “affordance” (Osiurak et al., 2017) and has been demonstrated using a variety of imaging techniques, including fMRI (Grafton et al., 1997; Grèzes et al., 2003). Since the reaction task generally necessitates a rapid reaction, such an experimental condition may promote the entire reaction.

Regarding the accuracy of the response, the “Hand and Fast” combination was observed to be significantly less accurate than the other combinations. Classical studies by Fitts (1954), Woodworth (1899), and others have shown a trade-off between speed and accuracy during exercise (Plamondon and Alimi, 1997). Even in a language processing task, it has been shown that errors increase when the go signal is issued immediately before the reaction (Mirabella et al., 2012). In other words, shorter word processing time increases the likelihood of an error occurrence. This study results can form the basis for suggesting that there is a trade-off between speed and accuracy, not only in behavior, but also at the cognitive level.

In stimulus set B, we used the onomatopoeia “Satto,” synonymous with “Quick,” to test whether the onomatopoeia facilitates a physical response. The onomatopoeia “Satto,” frequently used in motion guidance, had a significantly faster reaction time than “Quick,” even though no interaction effect was observed. Thus, “Satto” is more likely to promote action than “Quick.” Many studies have consistently demonstrated the benefits of symbolism in language learning, that is, using seemingly non-arbitrary linguistic or gestural cues that are symbolically linked to the word’s meaning (Imai et al., 2008; Asano et al., 2015; Lockwood et al., 2016). Onomatopoeia is believed to be present in most languages globally, and psychological experiments have shown that sound symbols in one language can be understood by speakers of another language (Svantesson, 2017). An fMRI study reported the involvement of the right posterior superior temporal sulcus, which is thought to be useful for understanding non-verbal communication, such as sound, in understanding onomatopoeia (Kanero et al., 2014). Thus, onomatopoeia is a word that can be understood through its sensory (e.g., auditory) features, although its exact meaning may be difficult to understand. This was confirmed by our study findings, wherein 54% of the participants indicated that “Satto” was easier to understand, as opposed to “Quick.” This provides a possible explanation for the absence of interaction effect observed in the RTs between “Quick” and “Satto.” Of particular interest is the finding that the NoE for “Foot and Satto” was lower than that observed for “Hand and Satto.” No previous studies have shown that body parts affect the understanding of language and onomatopoeia. This result indicates that the understanding of onomatopoeia may be influenced by attributes, such as body parts and the subject’s experience.

The current study has a few limitations. First, it was unclear whether ACE can also be observed when participants are instructed to react to the stimuli using the foot. Second, usual movement instruction is often carried out through auditory means; however, this study adopted visual stimulus presentation. Hence, the relationship between auditory understanding and behavior has not been clarified. Third, our study focused only on the onomatopoeia “Satto,” which limits the generalizability of the finding to other onomatopoeia terms. Future research will need to develop protocols that involve the use of auditory stimuli and foot responses. In addition, it is necessary to improve the matching of different terms to body parts through prior investigation of the onomatopoeia used for movement.

## Conclusion

We examined the effect of adverbs used in motion instruction on motion simulation and comprehension. “Fast” was found to promote motor responses, but can reduce language comprehension, leading to increased errors. The onomatopoeia “Satto” encourages more motor response than “Quick;” however, the degree of understanding of words may be affected by body parts. Therefore, it is necessary to confirm the participant’s comprehension level of a word in movement guidance.

## Data Availability Statement

The original contributions presented in the study are included in the article/Supplementary Material, further inquiries can be directed to the corresponding author.

## Ethics Statement

The studies involving human participants were reviewed and approved by the Kyoto University Graduate School and Faculty of Medicine (approval number R2188-1). The patients/participants provided their written informed consent to participate in this study.

## Author Contributions

KI and SZ designed the study. KI performed the experiment, analyzed the data, and wrote the manuscript. KI, KO, and NL interpreted the results. SZ checked the text and created part of the figure. NL checked and revised the manuscript. All authors approved the final version of the manuscript.

## Conflict of Interest

The authors declare that the research was conducted in the absence of any commercial or financial relationships that could be construed as a potential conflict of interest.

## Publisher’s Note

All claims expressed in this article are solely those of the authors and do not necessarily represent those of their affiliated organizations, or those of the publisher, the editors and the reviewers. Any product that may be evaluated in this article, or claim that may be made by its manufacturer, is not guaranteed or endorsed by the publisher.
