# Supplementary material for: Examining the Effect of Adverbs and Onomatopoeia on Physical Movement
Source: Front Psychol. 2021 Sep 22;12:723602. doi: 10.3389/fpsyg.2021.723602 (PMC8492989; doi:10.3389/fpsyg.2021.723602)
Supplement: Supplementary file 1 [file Table_1.docx]

Supplementary Material

**Supplemental Table 1. List of stimulus sets, Part A.**

| Original (Hand) | Fast | Slow |
| --- | --- | --- |
| Brush your teeth  歯を磨く | Brush your teeth faster  歯をすばやく磨く | Brush your teeth slowly  歯をゆっくり磨く |
| Unlock the door  鍵を開ける | Unlock the door faster  鍵をすばやく開ける | Unlock the door slowly  鍵をゆっくり開ける |
| Unbutton clothes  ボタンを外す | Unbutton clothes faster  ボタンをすばやく外す | Unbutton clothes slowly  ボタンをゆっくり外す |
| Wash your face  顔を洗う | Wash your face faster  顔をすばやく洗う | Wash your face slowly  顔をゆっくり洗う |
| Wipe the table  テーブルを拭く | Wipe the table faster  テーブルをすばやく拭く | Wipe the table slowly  テーブルをゆっくり拭く |
| Open the drawer  引き出しを開ける | Open the drawer faster  引き出しをすばやく開ける | Open the drawer slowly  引き出しをゆっくり開ける |
| Open the door  扉を開ける | Open the door faster  扉をすばやく開ける | Open the door slowly  扉をゆっくり開ける |
| Hang up the receiver  受話器を切る | Hang up the receiver faster  受話器をすばやく切る | Hang up the receiver slowly  受話器をゆっくり切る |
| Eat bread  パンを食べる | Eat bread faster  パンをすばやく食べる | Eat bread slowly  パンをゆっくり食べる |
| Drink water  水を飲む | Drink water faster  水をすばやく飲む | Drink water slowly  水をゆっくり飲む |
| Wear the clothes  服を着る | Wear the clothes faster  服をすばやく着る | Wear the clothes slowly  服をゆっくり着る |
| Pick up coins  コインを拾う | Pick up coins faster  コインをすばやく拾う | Pick up coins slowly  コインをゆっくり拾う |
| Pour water  水を注ぐ | Pour water faster  水をすばやく注ぐ | Pour water slowly  水をゆっくり注ぐ |
| Open the lid  蓋を開ける | Open the lid faster  蓋をすばやく開ける | Open the lid slowly  蓋をゆっくり開ける |
| Cut apples  リンゴを切る | Cut apples faster  リンゴをすばやく切る | Cut apples slowly  リンゴをゆっくり切る |
| Hang out the laundry  洗濯物を干す | Hang out the laundry faster  洗濯物をすばやく干す | Hang out the laundry slowly  洗濯物をゆっくり干す |
| Wash hair  髪を洗う | Wash hair faster  髪をすばやく洗う | Wash hair slowly  髪をゆっくり洗う |
| Flip the page  ページをめくる | Flip the page faster  ページをすばやくめくる | Flip the page slowly  ページをゆっくりめくる |
| Write a letter  手紙を書く | Write a letter faster  手紙をすばやく書く | Write a letter slowly  手紙をゆっくり書く |
| Draw a picture | Draw a picture faster | Draw a picture slowly |
| 絵を描く | 絵をすばやく描く | 絵をゆっくり描く |
| Original (Foot) | Fast | Slow |
| Stand up  立ち上がる | Stand up faster  すばやく立ちあがる | Stand up slowly  ゆっくり立ち上がる |
| Stand on toes  つま先で立つ | Stand on toes faster  つま先ですばやく立つ | Stand on toes slowly  つま先でゆっくり立つ |
| Raise your toes  足を挙げる | Raise your toes faster  足をすばやく挙げる | Raise your toes slowly  足をゆっくり挙げる |
| Open your legs  両足を開く | Open both legs faster  両足をすばやく開く | Open both legs slowly  両足をゆっくり開く |
| Kick the ball  ボールを蹴る | Kick the ball faster  ボールをすばやく蹴る | Kick the ball slowly  ボールをゆっくり蹴る |
| Step on  乗る | Step on faster  すばやく乗る | Step on slowly  ゆっくり乗る |
| Climb the stairs  階段を上る | Climb the stairs faster  階段を素早く上る | Climb the stairs slowly  階段をゆっくり上る |
| Go down the stairs  階段を下る | Go down the stairs faster  階段をすばやく下る | Go down the stairs slowly  階段をゆっくり下る |
| Turn the pedal  ペダルを漕ぐ | Turn the pedal faster  ペダルをすばやく漕ぐ | Turn the pedal slowly  ペダルをゆっくり漕ぐ |
| Sit down  座る | Sit down chair faster  椅子にすばやく座る | Sit down chair slowly  椅子にゆっくり座る |
| Cross your legs  両足を組む | Cross your legs faster  両足をすばやく組む | Cross your legs slowly  両足をゆっくり組む |
| Run  走る | Run in the courtyard faster  中庭をすばやく走る | Run in the courtyard slowly  中庭をゆっくり走る |
| Jump  飛ぶ | Jump up faster  すばやく飛ぶ | Jump up slowly  ゆっくり飛ぶ |
| Jump off  飛び降りる | Jump off faster  すばやく飛び降りる | Jump off slowly  ゆっくり飛び降りる |
| Take steps  ステップを踏む | Take steps faster  ステップをすばやく踏む | Take steps slowly  ステップをゆっくり踏む |
| Raise one leg  片足を挙げる | Raise one leg faster  片足をすばやく挙げる | Raise one leg slowly  片足をゆっくり挙げる |
| Put on shoes  靴を履く | Put on shoes faster  靴をすばやく履く | Put on shoes slowly  靴をゆっくり履く |
| Close your legs  両足を閉じる | Close your legs faster  両足をすばやく閉じる | Close your legs slowly  両足をゆっくり閉じる |
| Walk  歩く | Walk on the pedestrian crossing faster  横断歩道をすばやく歩く | Walk on the pedestrian crossing slowly  横断歩道をゆっくり歩く |
| Bend your knees  膝を曲げる | Bend your knees faster  膝をすばやく曲げる | Bend your knees slowly  膝をゆっくり曲げる |

**Supplemental Table 2. List of stimulus sets, Part** B**.**

| Original (Hand) | Quick | Satto (in a flash) |
| --- | --- | --- |
| Brush your shoes  靴を磨く | Brush your shoes quickly  靴をはやく磨く | Brush your shoes in a flash  靴をさっと磨く |
| Lock the door  鍵を閉める | Lock the door quickly  鍵をはやく閉める | Lock the door in a flash  鍵をさっと閉める |
| Button clothes  ボタンを留める | Button clothes quickly  ボタンをはやく留める | Button clothes in a flash  ボタンをさっと留める |
| Wash your hand  手を洗う | Wash your hand quickly  手をはやく洗う | Wash your hand in a flash  手をさっと洗う |
| Wipe the table  テーブルを拭く | Wipe the table quickly  テーブルをはやく拭く | Wipe the table in a flash  テーブルをさっと拭く |
| Close the drawer  引き出しを閉める | Close the drawer quickly  引き出しをはやく閉める | Close the drawer in a flash  引き出しをさっと閉める |
| Close the door  扉を閉める | Close the door quickly  扉をはやく閉める | Close the door in a flash  扉をはやく閉める |
| Hang up the receiver  受話器を切る | Hang up the receiver quickly  受話器をはやく切る | Hang up the receiver in a flash  受話器をさっと切る |
| Eat rice  米を食べる | Eat rice quickly  米をはやく食べる | Eat rice in a flash  米をさっと食べる |
| Drink milk  牛乳を飲む | Drink milk quickly  牛乳をはやく飲む | Drink milk in a flash  牛乳をさっと飲む |
| Wear the clothes  服を着る | Wear the clothes quickly  服をはやく着る | Wear the clothes in a flash  服をさっと着る |
| Pick up coins  コインを拾う | Pick up coins quickly  コインをはやく拾う | Pick up coins in a flash  コインをさっと拾う |
| Pour water  水を注ぐ | Pour water quickly  水をはやく注ぐ | Pour water in a flash  水をさっと注ぐ |
| Open the lid  蓋を開ける | Open the lid quickly  蓋をはやく開ける | Open the lid in a flash  蓋をさっと開ける |
| Cut apples  リンゴを切る | Cut apples quickly  リンゴをはやく切る | Cut apples in a flash  リンゴをさっと切る |
| Taking in the laundry  洗濯物を取り込む | Taking in the laundry quickly  洗濯物をはやく取りこむ | Taking in the laundry in a flash  洗濯物をさっと取り込む |
| Wash arm  腕を洗う | Wash arm quickly  腕をはやく洗う | Wash arm in a flash  腕をさっと洗う |
| Flip the page  ページをめくる | Flip the page quickly  ページをはやくめくる | Flip the page in a flash  ページをさっとめくる |
| Write a letter  字を書く | Write a letter quickly  字をはやく書く | Write a letter in a flash  字をさっと書く |
| Draw a picture | Draw a picture quickly | Draw a picture in a flash |
| 絵を描く | 絵をはやく描く | 絵をさっと描く |
| Original (Foot) | Quick | Satto (in a flash) |
| Stand up  立ち上がる | Stand up quickly  はやく立ちあがる | Stand up in a flash  さっと立ち上がる |
| Stand on toes  つま先で立つ | Stand on toes quickly  つま先ではやく立つ | Stand on toes in a flash  つま先でさっと立つ |
| Raise your toes  足を挙げる | Raise your toes quickly  足をはやく挙げる | Raise your toes in a flash  足をさっと挙げる |
| Open your legs  両足を開く | Open both legs quickly  両足をはやく開く | Open both legs in a flash  両足をさっと開く |
| Kick the ball  ボールを蹴る | Kick the ball quickly  ボールをはやく蹴る | Kick the ball in a flash  ボールをさっと蹴る |
| Step on  乗る | Step on quickly  はやく乗る | Step on in a flash  さっと乗る |
| Climb the stairs  階段を上る | Climb the stairs quickly  階段を素早く上る | Climb the stairs in a flash  階段をさっと上る |
| Go down the stairs  階段を下る | Go down the stairs quickly  階段をはやく下る | Go down the stairs in a flash  階段をさっと下る |
| Turn the pedal  ペダルを漕ぐ | Turn the pedal quickly  ペダルをはやく漕ぐ | Turn the pedal in a flash  ペダルをさっと漕ぐ |
| Sit down  座る | Sit down on the chair quickly  椅子にはやく座る | Sit down on a chair in a flash  椅子にさっと座る |
| Cross your legs  両足を組む | Cross your legs quickly  両足をはやく組む | Cross your legs in a flash  両足をさっと組む |
| Run  走る | Run in the courtyard quickly  中庭をはやく走る | Run in the courtyard in a flash  中庭をさっと走る |
| Jump  飛ぶ | Jump up quickly  はやく飛ぶ | Jump up in a flash  さっと飛ぶ |
| Jump off  飛び降りる | Jump off quickly  はやく飛び降りる | Jump off in a flash  さっと飛び降りる |
| Take steps  ステップを踏む | Take steps quickly  ステップをはやく踏む | Take steps in a flash  ステップをさっと踏む |
| Raise one leg  片足を挙げる | Raise one leg quickly  片足をはやく挙げる | Raise one leg in a flash  片足をさっと挙げる |
| Put on shoes  靴を履く | Put on shoes quickly  靴をはやく履く | Put on shoes in a flash  靴をさっと履く |
| Close your legs  両足を閉じる | Close your legs quickly  両足をはやく閉じる | Close your legs in a flash  両足をさっと閉じる |
| Walk  歩く | Walk on the pedestrian crossing quickly  横断歩道をはやく歩く | Walk on the pedestrian crossing in a flash  横断歩道をさっと歩く |
| Bend your knees  膝を曲げる | Bend your knees quickly  膝をはやく曲げる | Bend your knees in a flash  膝をさっと曲げる |
